# Supplementary figures and images for: Amino-acid site variability among natural and designed proteins
Source: PeerJ. 2013 Nov 12;1:e211. doi: 10.7717/peerj.211 (PMC3828621; doi:10.7717/peerj.211)

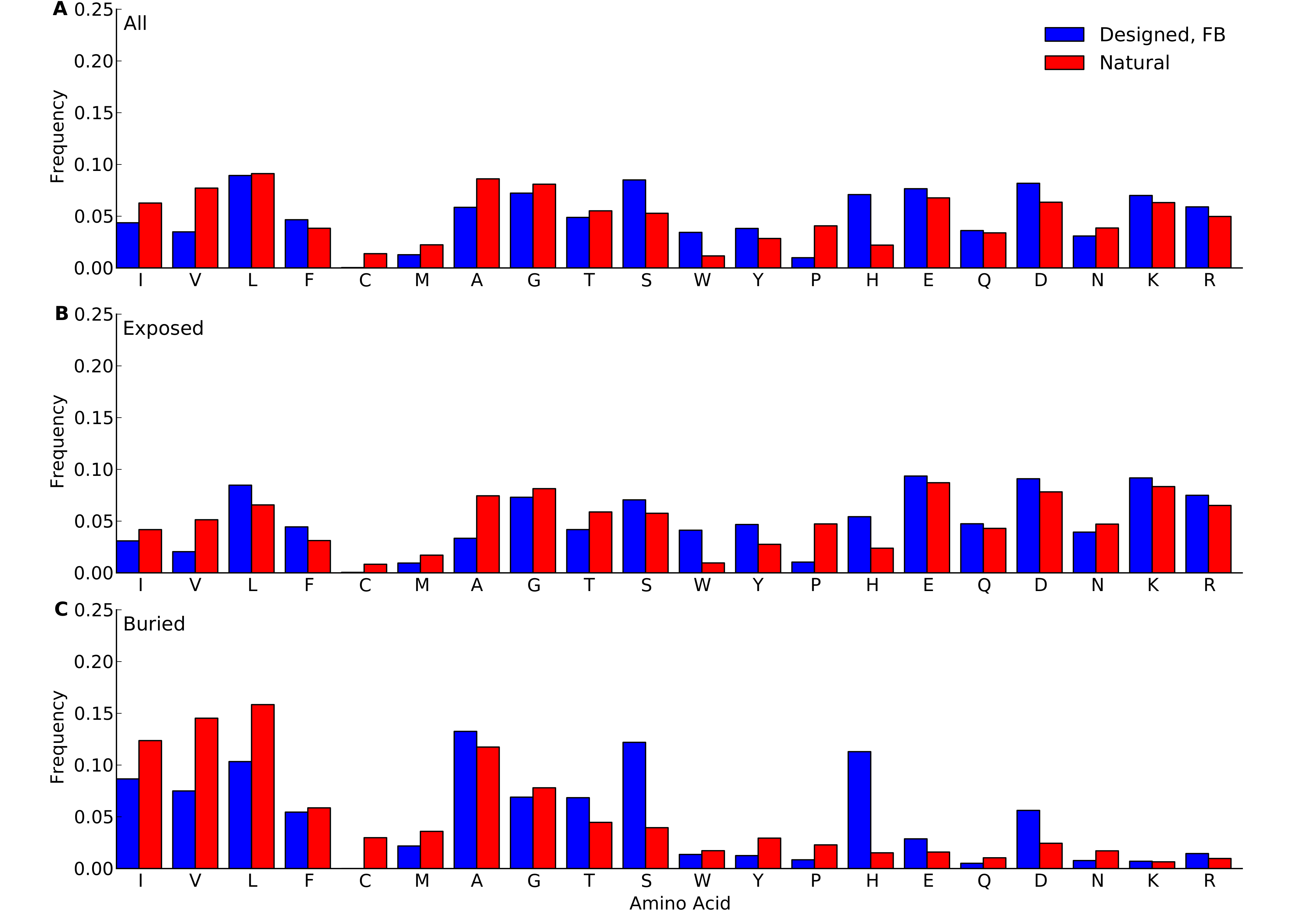

Supplement: Figure S1 — Frequencies were calculated over all sites in all proteins belonging to the yeast-proteins data set. For designed proteins, only fixed-backbone designs were considered. (A) overall frequencies. (B) frequencies at exposed sites (defined as sites with RSA > 0.05). (C) frequencies at buried sites (defined as sites with RSA ≤ 0.05). [file peerj-01-211-s001.png]

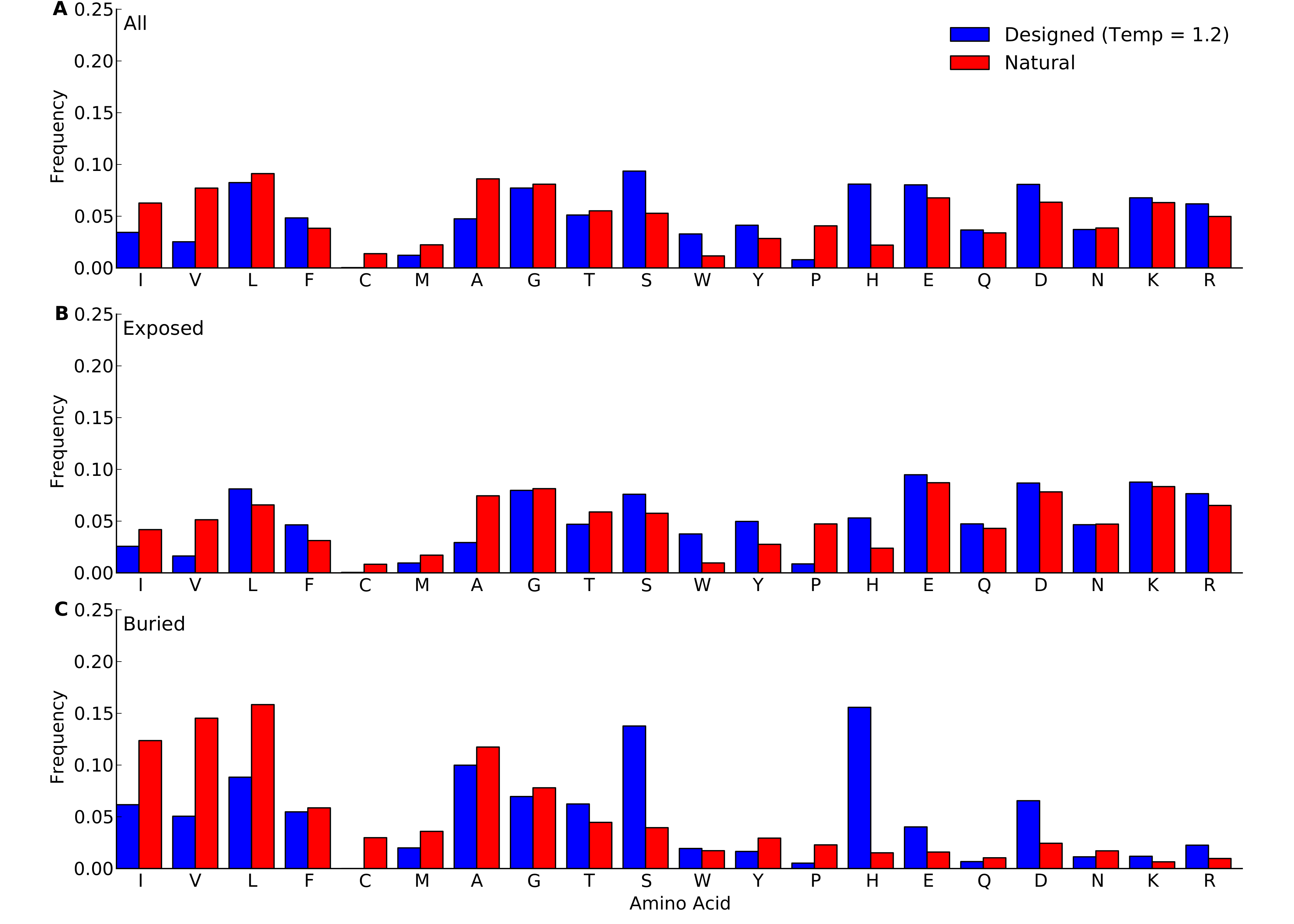

Supplement: Figure S2 — Frequencies were calculated over all sites in all proteins belonging to the yeast-proteins data set. For designed proteins, only flexible-backbone designs with design temperature 1.2 were considered. (A) overall frequencies. (B) frequencies at exposed sites (defined as sites with RSA > 0.05). (C) frequencies at buried sites (defined as sites with RSA ≤ 0.05). [file peerj-01-211-s002.png]

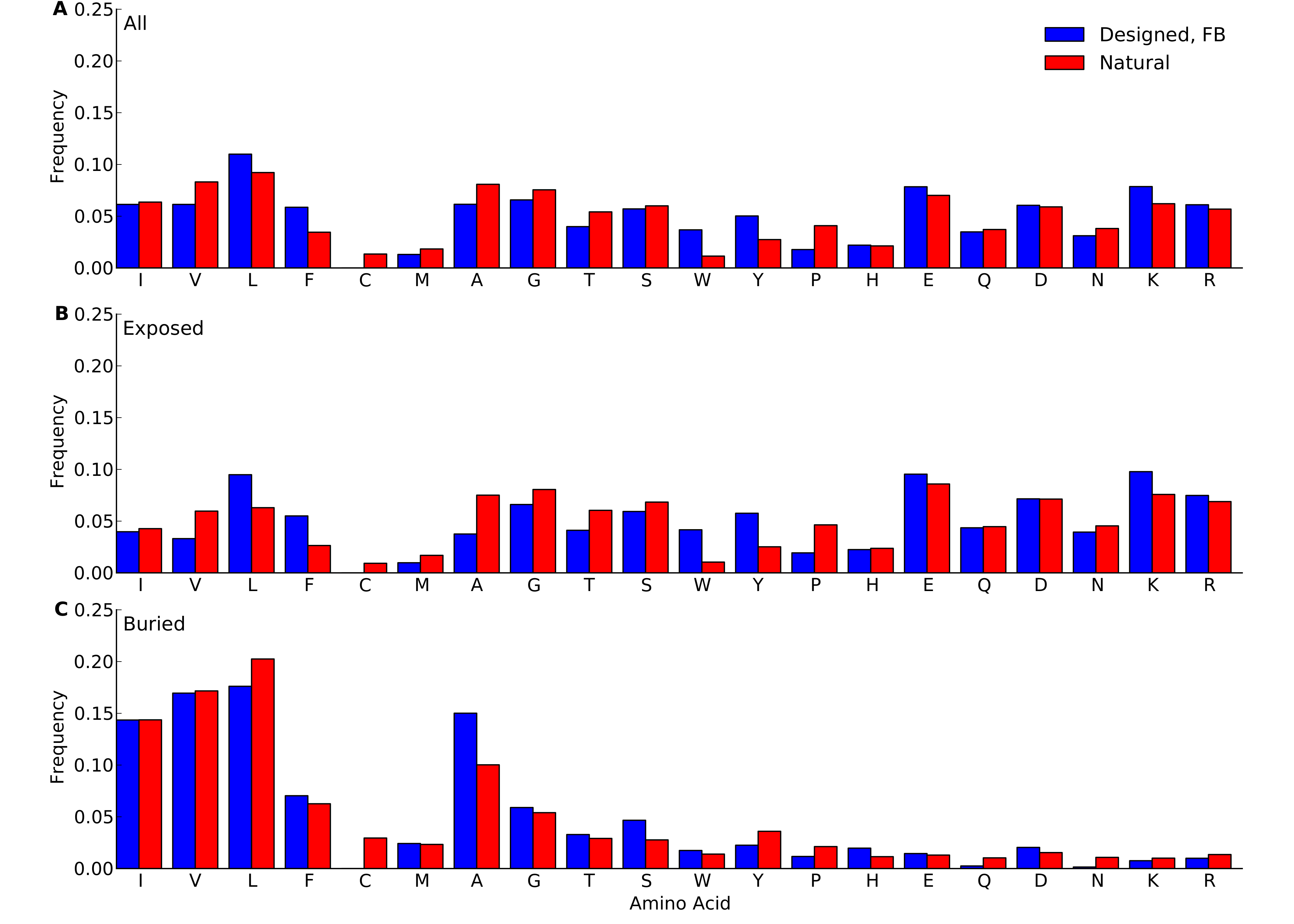

Supplement: Figure S3 — Frequencies were calculated over all sites in all proteins belonging to the protein-domains data set. For designed proteins, only fixed-backbone designs were considered. (A) overall frequencies. (B) frequencies at exposed sites (defined as sites with RSA > 0.05). (C) frequencies at buried sites (defined as sites with RSA ≤ 0.05). [file peerj-01-211-s003.png]

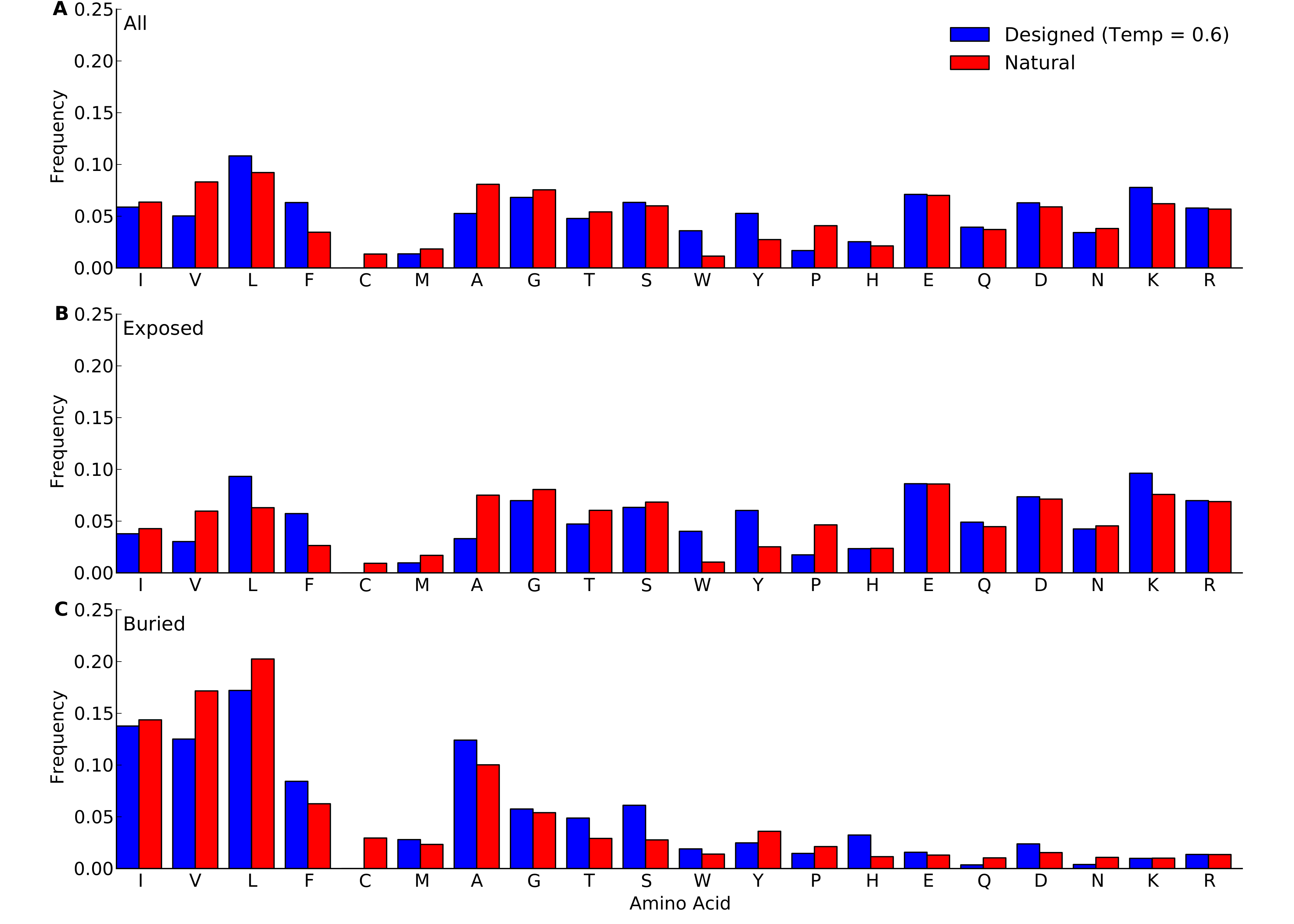

Supplement: Figure S4 — Frequencies were calculated over all sites in all proteins belonging to the protein-domains data set. For designed proteins, only flexible-backbone designs with design temperature 0.6 were considered. (A) overall frequencies. (B) frequencies at exposed sites (defined as sites with RSA > 0.05). (C) frequencies at buried sites (defined as sites with RSA ≤ 0.05). [file peerj-01-211-s004.png]

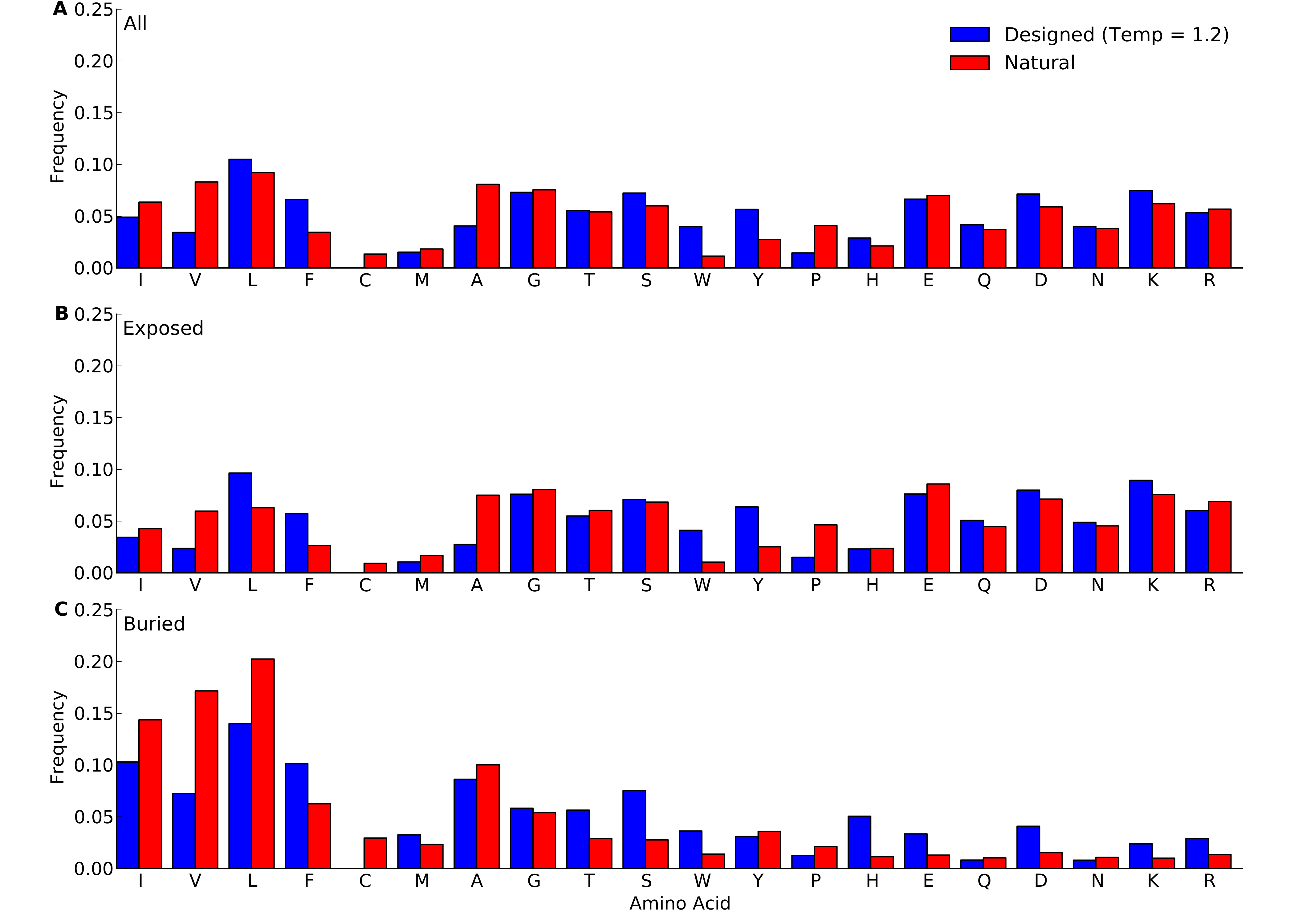

Supplement: Figure S5 — Frequencies were calculated over all sites in all proteins belonging to the protein-domains data set. For designed proteins, only flexible-backbone designs with design temperature 1.2 were considered. (A) overall frequencies. (B) frequencies at exposed sites (defined as sites with RSA > 0.05). (C) frequencies at buried sites (defined as sites with RSA ≤ 0.05). [file peerj-01-211-s005.png]

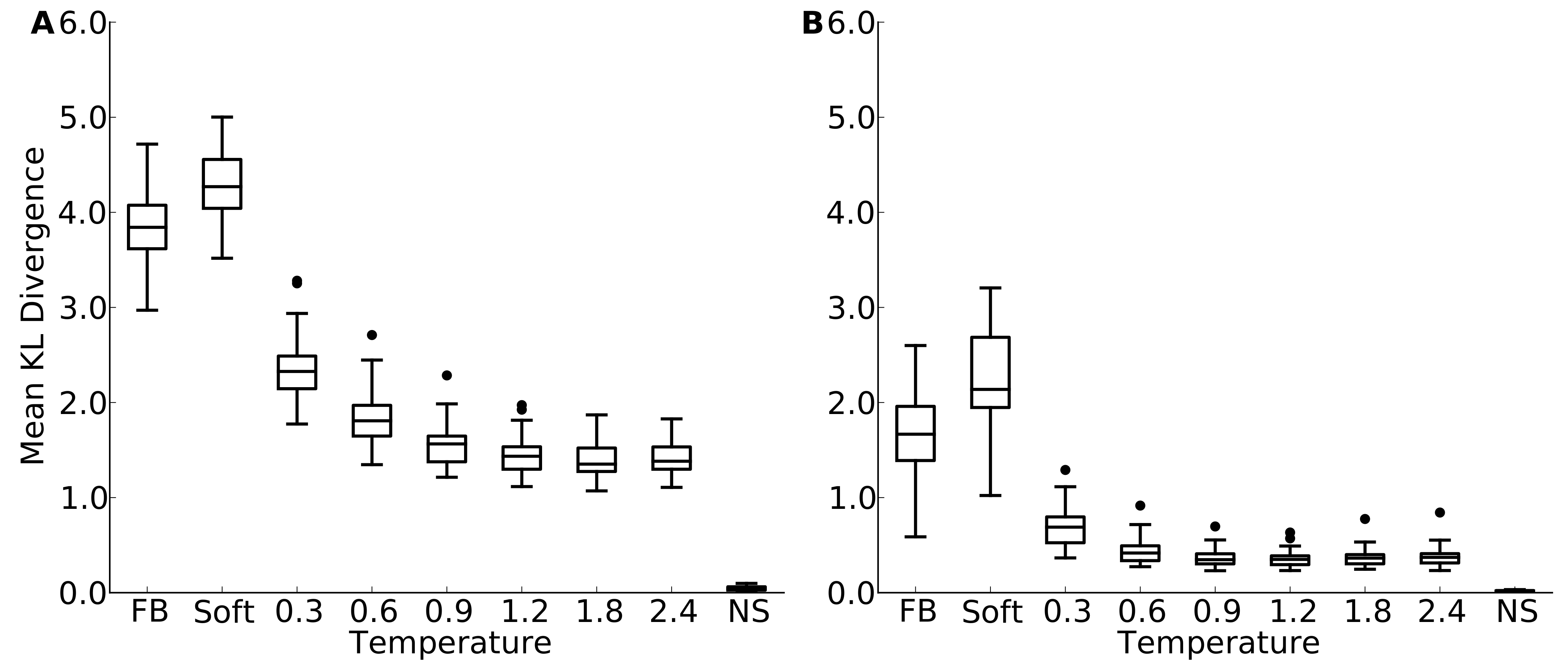

Supplement: Figure S6 — A higher KL divergence indicates that the amino-acid distributions at sites in designed proteins are less similar to the corresponding distributions in the natural proteins. “FB” refers to fixed backbone design, and “NS” refers to the control case where natural sequences are compared to themselves. (A) KL divergence calculated from the relative frequencies of the 20 amino acids. (B) KL divergence calculated from rank-ordered frequency distributions. The most common amino acid in the reference distribution is compared to the most common amino acid in the focal distribution, the same is done for the second-most common amino acid, and so on, irrespective of the type of amino acids. [file peerj-01-211-s006.png]

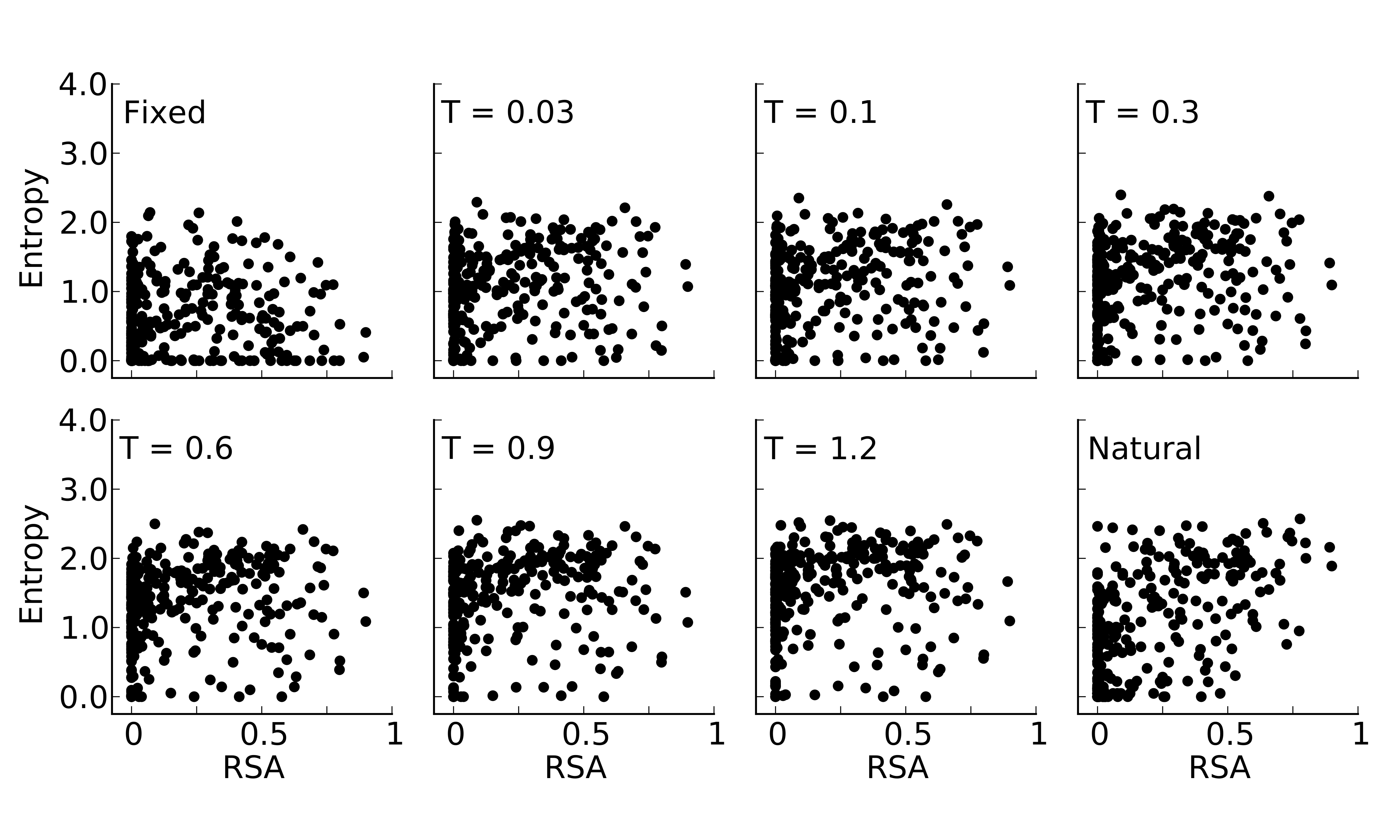

Supplement: Figure S7 — Natural sequences exhibit a clear trend of higher site variability at higher RSA values. The flexible backbone designs exhibit a similar trend but the fixed backbone designs do not. [file peerj-01-211-s007.png]

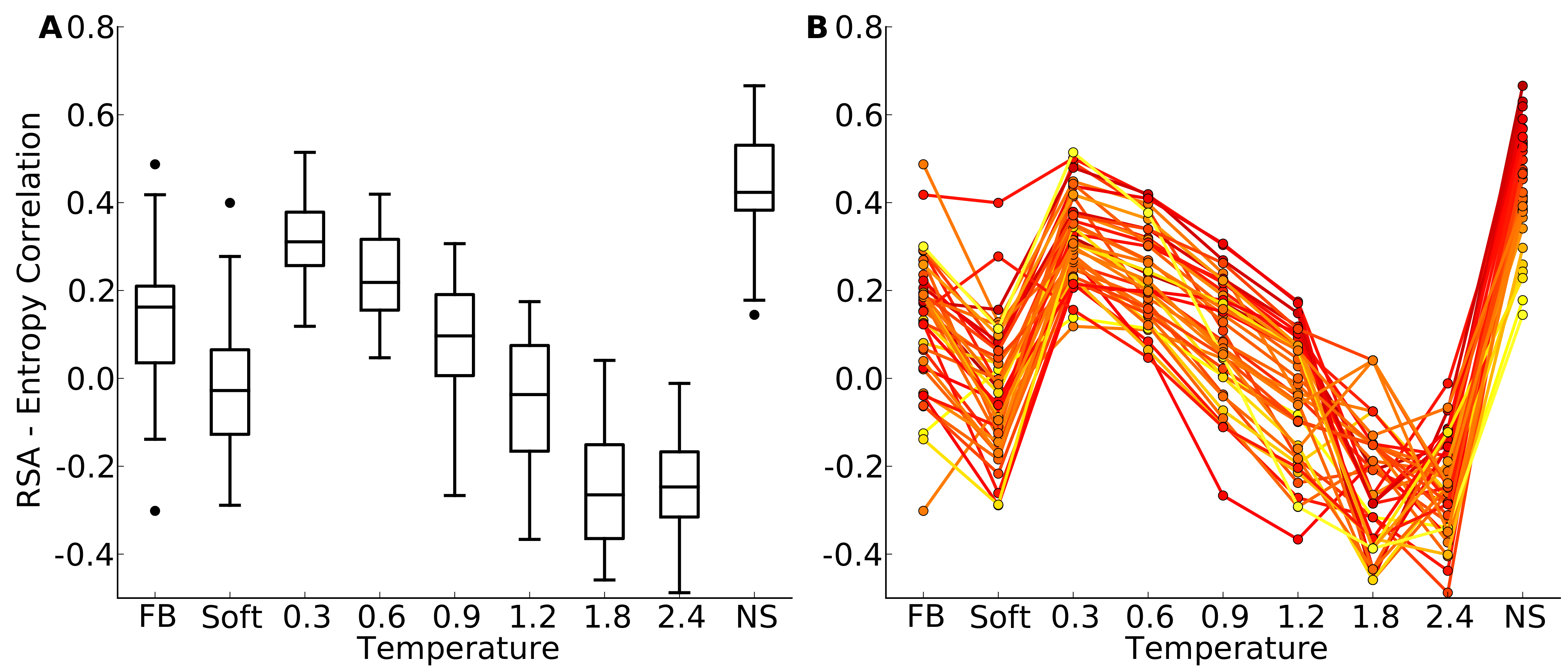

Supplement: Figure S8 — “FB” indicates fixed-backbone design, “Soft” indicates soft backbone design, and “NS” indicates natural sequences. (A) Distributions represented as boxplots. (B) Correlation coefficients for individual proteins. Lines connect identical structures in the different design conditions. The color shading represents the strength of the correlation for the natural sequence alignment. In general, natural proteins display a stronger correlation between site entropy and RSA than designed proteins. [file peerj-01-211-s008.png]

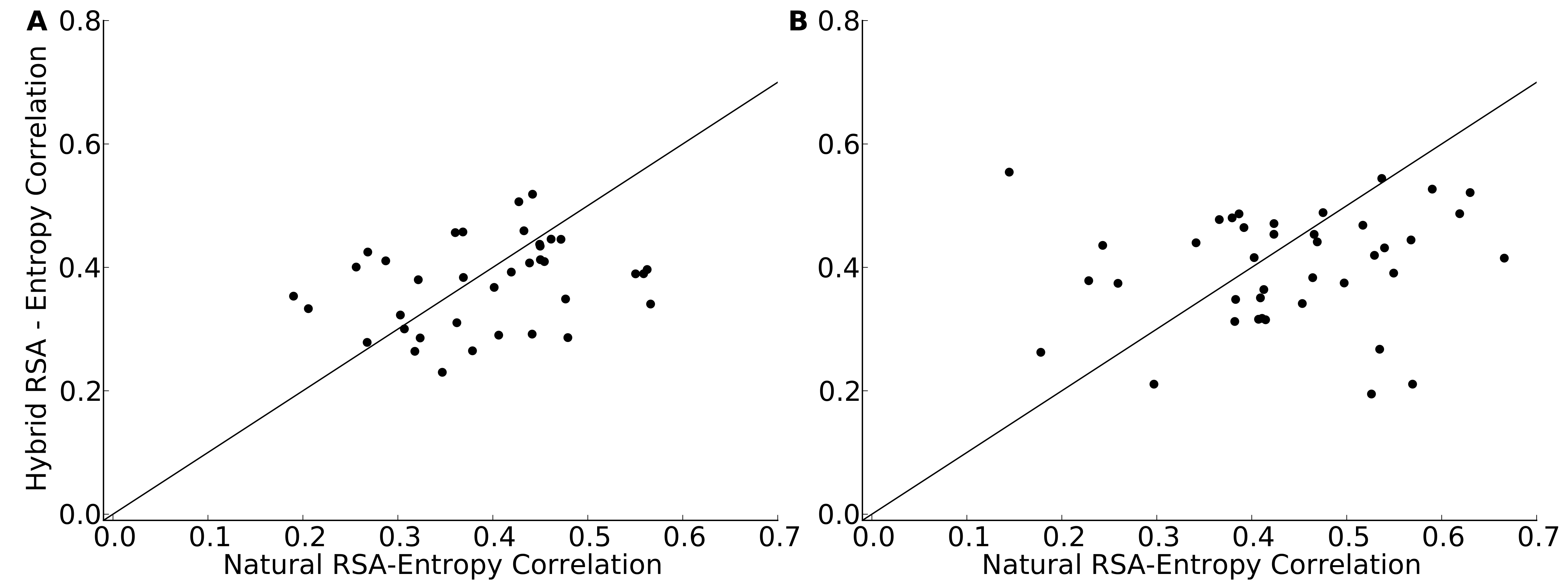

Supplement: Figure S9 — For the hybrid designs, buried and partially buried sites were taken from proteins designed with a fixed backbone (yeast proteins) or a temperature of T = 0.6 (protein domains). Exposed residues were taken from proteins designed with a temperature of T = 0.1 (yeast proteins) or T = 1.8 (protein domains). The solid line indicates y = x. Note that while the range of correlation values in hybrid designs generally matches the range of values in natural alignments, predictions for specific proteins are not that accurate. [file peerj-01-211-s009.png]
